# Supplementary material for: Causal Relationship between Aging and Anorexia Nervosa: A White-Matter-Microstructure-Mediated Mendelian Randomization Analysis
Source: Biomedicines. 2024 Aug 16;12(8):1874. doi: 10.3390/biomedicines12081874 (PMC11351342; doi:10.3390/biomedicines12081874)
Supplement: Supplementary file 1 [file biomedicines-12-01874-s001.zip › Supplementary Figure/Figure S4/Singe SNP plot of MR analysis for TL to ubm-b-1930.pdf]

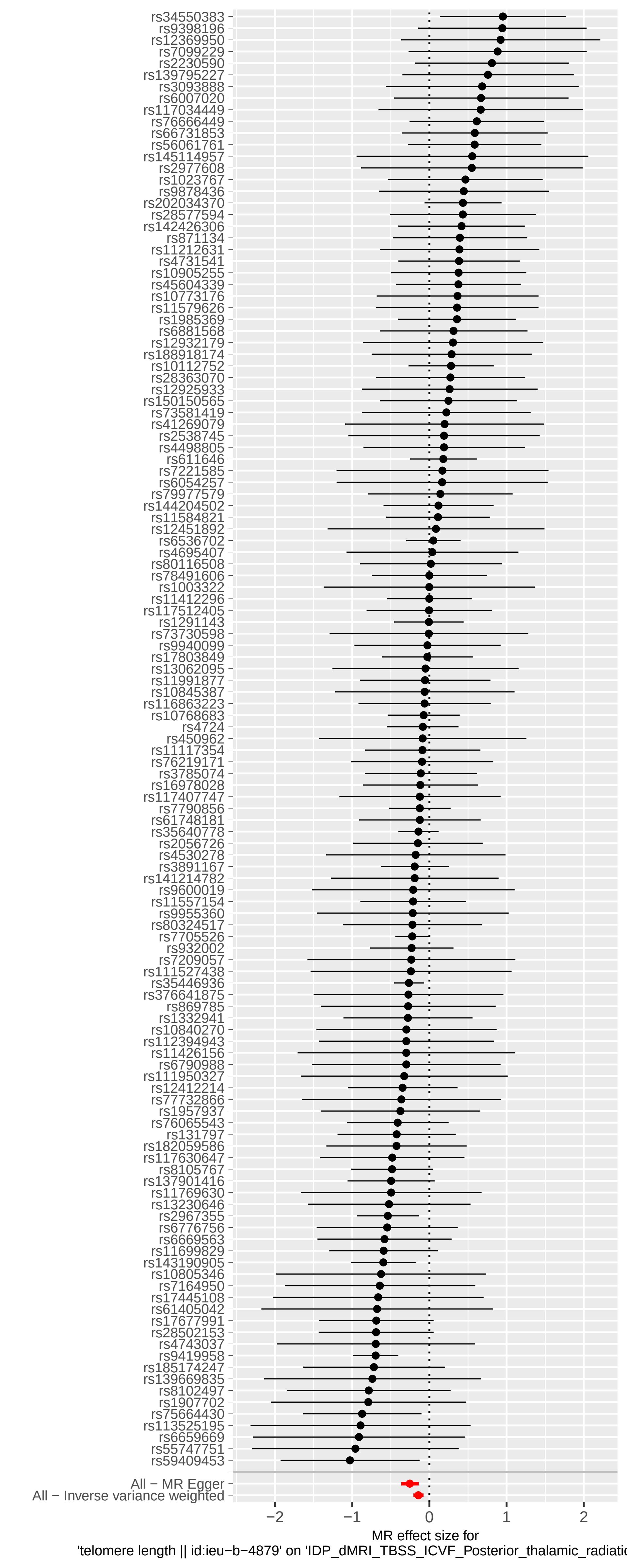
rs34550383
rs9398196
rs12369950
rs7099229
rs2230590
rs139795227
rs3093888
rs6007020
rs117034449
rs76666449
rs66731853
rs56061761
rs145114957
rs2977608
rs1023767
rs9878436
rs202034370
rs28577594
rs142426306
rs871134
rs11212631
rs4731541
rs10905255
rs45604339
rs10773176
rs11579626
rs1985369
rs6881568
rs12932179
rs188918174
rs10112752
rs28363070
rs12925933
rs150150565
rs73581419
rs41269079
rs2538745
rs4498805
rs611646
rs7221585
rs6054257
rs79977579
rs144204502
rs11584821
rs12451892
rs6536702
rs4695407
rs80116508
rs78491606
rs1003322
rs11412296
rs117512405
rs1291143
rs73730598
rs9940099
rs17803849
rs13062095
rs11991877
rs10845387
rs116863223
rs10768683
rs4724
rs450962
rs11117354
rs76219171
rs3785074
rs16978028
rs117407747
rs7790856
rs61748181
rs35640778
rs2056726
rs4530278
rs3891167
rs141214782
rs9600019
rs11557154
rs9955360
rs80324517
rs7705526
rs932002
rs7209057
rs111527438
rs35446936
rs376641875
rs869785
rs1332941
rs10840270
rs112394943
rs11426156
rs6790988
rs111950327
rs12412214
rs77732866
rs1957937
rs76065543
rs131797
rs182059586
rs117630647
rs8105767
rs137901416
rs11769630
rs13230646
rs2967355
rs6776756
rs6669563
rs11699829
rs143190905
rs10805346
rs7164950
rs17445108
rs61405042
rs17677991
rs28502153
rs4743037
rs9419958
rs185174247
rs139669835
rs8102497
rs1907702
rs75664430
rs113525195
rs6659669
rs55747751
rs59409453
All − MR Egger
All − Inverse variance weighted
−2
−1
0
1
2
MR effect size for
'telomere length || id:ieu−b−4879' on 'IDP_dMRI_TBSS_ICVF_Posterior_thalamic_radiatio
